# 9-(2-Hy­droxy-6-oxocyclo­hex-1-en-1-yl)-2,3,4,9-tetra­hydro-1*H*-xanthen-1-one

**DOI:** 10.1107/S1600536813007952

**Published:** 2013-03-28

**Authors:** Shaaban K. Mohamed, Mehmet Akkurt, Antar A. Abdelhamid, Aamer Saeed, Ulrich Flörke

**Affiliations:** a Chemistry and Environmental Division, Manchester Metropolitan University, Manchester M1 5GD, England; b Department of Physics, Faculty of Sciences, Erciyes University, 38039 Kayseri, Turkey; c Department of Chemistry, Quaid-i-Azam University, Islamabad 45320, Pakistan; d Department Chemie, Fakultät für Naturwissenschaften, Universität Paderborn, Warburgerstrasse 100, D-33098 Paderborn, Germany

## Abstract

In the xanthene ring system in the title compound, C_19_H_18_O_4_, the 4*H*-pyran ring has a maximum deviation of 0.110 (2) Å from planarity and the cyclo­hexene ring exhibits a puckered conformation [puckering parameters *Q*_T_ = 0.452 (3) Å, θ = 57.0 (4) and ϕ = 131.7 (4)°]. The cyclo­hexene ring attached to the xanthene system adopts an envelope conformation, with the middle of the three methylene C atoms as the flap atom. In the crystal, O—H⋯O and C—H⋯O hydrogen bonds form infinite chains of *R*_1_^2^(6) ring motifs along [100] with the xanthene groups arranged in an alternating zigzag manner.

## Related literature

For the bioactivity of xanthene compounds, see: Mohamed *et al.* (2012*a* ▶); Mo *et al.* (2010 ▶) and for their fluorescence properties, see: Menchen *et al.* (2003 ▶). For similar structures see: Mohamed *et al.* (2011 ▶, 2012*b* ▶); Kurbanova *et al.* (2012 ▶); Abdelhamid *et al.* (2011 ▶); Reddy *et al.* (2009 ▶). For ring conformations, see: Cremer & Pople (1975 ▶). For hydrogen-bond motifs, see: Bernstein *et al.* (1995 ▶).
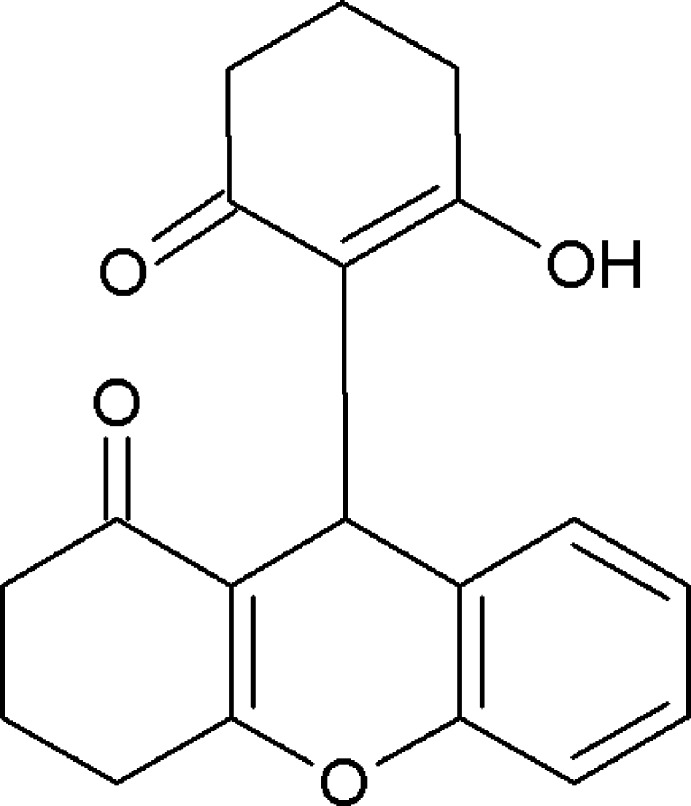


## Experimental

### 

#### Crystal data

C_19_H_18_O_4_
*M* *_r_* = 310.33
Orthorhombic, 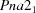

*a* = 13.4420 (18) Å
*b* = 8.0015 (10) Å
*c* = 14.2416 (18) Å
*V* = 1531.8 (3) Å^3^
*Z* = 4
Mo *K*α radiation
μ = 0.09 mm^−1^
*T* = 130 K
0.37 × 0.24 × 0.15 mm

#### Data collection

Bruker SMART APEX diffractometer
Absorption correction: multi-scan (*SADABS*; Sheldrick, 2004 ▶) *T*_min_ = 0.966, *T*_max_ = 0.986
13880 measured reflections
1901 independent reflections
1799 reflections with *I* > 2σ(*I*)
*R*_int_ = 0.032

#### Refinement

*R*[*F* ^2^ > 2σ(*F* ^2^)] = 0.043
*wR*(*F* ^2^) = 0.111
*S* = 1.06
1901 reflections
209 parameters
1 restraint
H-atom parameters constrained
Δρ_max_ = 0.39 e Å^−3^
Δρ_min_ = −0.18 e Å^−3^

### 

Data collection: *SMART* (Bruker, 2002 ▶); cell refinement: *SAINT* (Bruker, 2002 ▶); data reduction: *SAINT*; program(s) used to solve structure: *SHELXS97* (Sheldrick, 2008 ▶); program(s) used to refine structure: *SHELXL97* (Sheldrick, 2008 ▶); molecular graphics: *PLATON* (Spek, 2009 ▶); software used to prepare material for publication: *WinGX* (Farrugia, 2012 ▶) and *PLATON*.

## Supplementary Material

Crystal structure: contains datablock(s) global, I. DOI: 10.1107/S1600536813007952/nk2202sup1.cif

Structure factors: contains datablock(s) I. DOI: 10.1107/S1600536813007952/nk2202Isup2.hkl

Supplementary material file. DOI: 10.1107/S1600536813007952/nk2202Isup3.cml

Additional supplementary materials:  crystallographic information; 3D view; checkCIF report

## Figures and Tables

**Table 1:** Hydrogen-bond geometry (Å, °)

*D*—H⋯*A*	*D*—H	H⋯*A*	*D*⋯*A*	*D*—H⋯*A*
O2—H2⋯O1^i^	0.84	1.76	2.582 (2)	164
C5—H5*A*⋯O1^i^	0.99	2.42	3.034 (3)	119

Symmetry code: (i) 
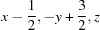
.

**Fig. 1.**
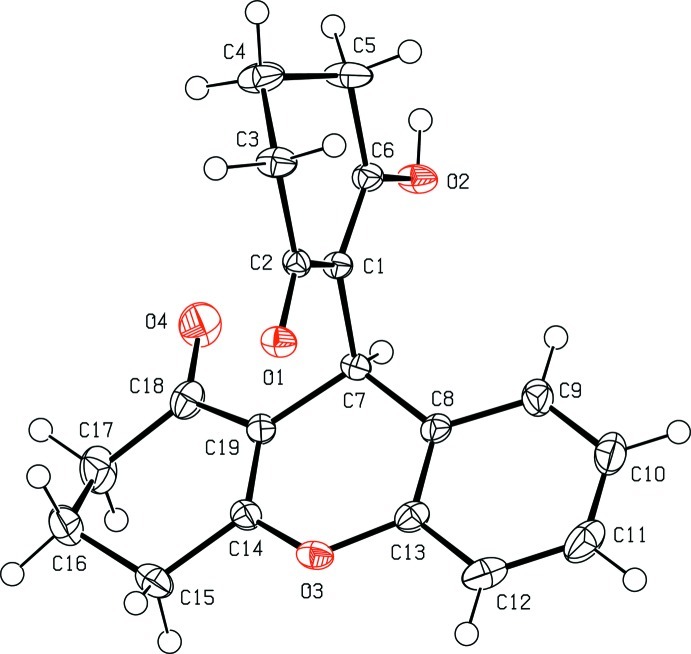
Molecular structure of title compound. Displacement ellipsoids are drawn at the 50% probability level.

**Fig. 2.**
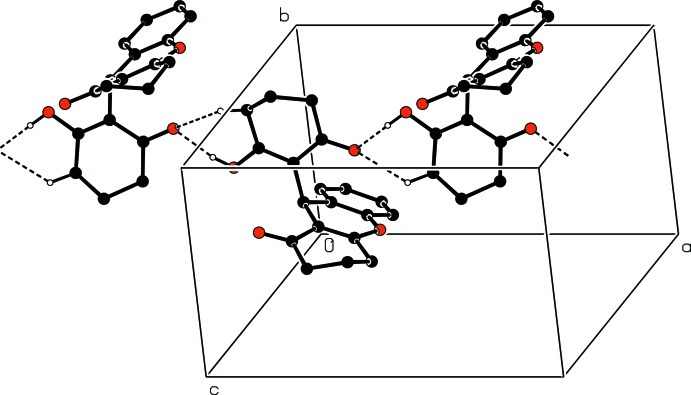
The intermolecular hydrogen bonds in (I) indicated as dashed lines along [001]. H-atoms not involved in hydrogen bonding are omitted.

## supplementary crystallographic information

### Comment

Due to the great spectroscopic and biological applications of xanthene molecules, they attracted an excessive interest of researchers in different fields of medicinal and applied chemistry. Such derivatives have exhibited fluorescence properties (Menchen *et al.*, 2003) in addition to their fungicidal, bactericidal and anti-inflammatory possessions (Mohamed *et al.*, 2012*a*; Mo *et al.*, 2010). As part of our on-going study on synthesis of potential biologically active molecules based xanthene core structure compounds, herein we report the synthesis and structural study of the title compound.

The title compound (I) is shown in Fig. 1. In the xanthene ring system (O3/C7–C19) of (I), the 4*H*-pyran ring (O3/C7/C8/C13/C14/C19) is nearly planar [maximum deviation = 0.110 (2) Å] and the cyclohexene ring (C14–C19) is puckered with the puckering parameters (Cremer & Pople, 1975) of *Q*_T_ = 0.452 (3) Å, θ = 57.0 (4) ° and φ = 131.7 (4) °. The cyclohexene ring (C1–C6) attached to the xanthene system adopts an envelope conformation with the puckering parameters of *Q*_T_ = 0.477 (3) Å, θ = 60.5 (2) ° and φ = 178.1 (3) °. The bond lengths and bond angles fall within a normal range and are comparable with those of the similar structures previously reported (Mohamed *et al.*, 2012*b*; Kurbanova *et al.*, 2012; Abdelhamid *et al.*, 2011; Mohamed *et al.*, 2011; Reddy *et al.*, 2009).

Intermolecular O2—H···O1^i^ and C5—H5A···O1^i^ [(i): *x* - 0.5, -*y* + 1.5, *z*; Table 1] hydrogen bonds form infinite chains of *R*^2^_1_(6)ring motifs (Bernstein *et al.*, 1995; Fig. 2) along the *a* axis with xanthen groups in alternating zigzag manner.

### Experimental

The title compound was obtained as a major product from a three component reaction of 112 mg (1 mmol) cyclohexane-1,3-dione, 112 mg(1 mmol)salicylaldehyde and 137 mg (1 mmol) 1-(3-aminophenyl)ethanol in 50 ml ethanol [the amino alcohol in this reaction has not been reacted instead acted as a Lewis base catalyst]. The reaction mixture was refluxed for 3 h at 350 K, then cooled at room temperature in fume cupboard where the excess solvent was evaporated. The solid that formed was filtered off, washed with cold ethanol and dried under vacuum. On crystallization from ethanol shiny crystals (m.p. 503 K) were collected in an excellent yield (92%). Crystals suitable for X-ray diffraction were grown by slow evaporation method over two days using ethanol solution.

### Refinement

All H atoms were positioned geometrically and refined as riding on their parent atoms with O—H = 0.84 Å, C—H = 0.95–1.00 Å and *U*_iso_(H) = 1.5*U*_eq_(O) for hydroxyl H or 1.2*U*_eq_(C) for other H atoms. The H atom of the hydroxyl group was placed using the rotating group refinement option (AFIX 147). Missing symmetry was checked using ADDSYM feature in *PLATON* (Spek, 2009). Friedel pairs were merged by using MERG 3 instruction.

### Crystal data

**Table:**

C_19_H_18_O_4_	*F*(000) = 656
*M**_r_* = 310.33	*D*_x_ = 1.346 Mg m^−^^3^
Orthorhombic, *P**n**a*2_1_	Mo *K*α radiation, λ = 0.71073 Å
Hall symbol: P 2c -2n	Cell parameters from 3393 reflections
*a* = 13.4420 (18) Å	θ = 2.9–27.9°
*b* = 8.0015 (10) Å	µ = 0.09 mm^−^^1^
*c* = 14.2416 (18) Å	*T* = 130 K
*V* = 1531.8 (3) Å^3^	Prism, pale-yellow
*Z* = 4	0.37 × 0.24 × 0.15 mm

### Data collection

**Table:**

Bruker SMART APEX diffractometer	1901 independent reflections
Radiation source: sealed tube	1799 reflections with *I* > 2σ(*I*)
Graphite monochromator	*R*_int_ = 0.032
φ and ω scans	θ_max_ = 27.9°, θ_min_ = 2.9°
Absorption correction: multi-scan (*SADABS*; Sheldrick, 2004)	*h* = −17→17
*T*_min_ = 0.966, *T*_max_ = 0.986	*k* = −10→10
13880 measured reflections	*l* = −18→18

### Refinement

**Table:**

Refinement on *F*^2^	Primary atom site location: structure-invariant direct methods
Least-squares matrix: full	Secondary atom site location: difference Fourier map
*R*[*F*^2^ > 2σ(*F*^2^)] = 0.043	Hydrogen site location: difference Fourier map
*wR*(*F*^2^) = 0.111	H-atom parameters constrained
*S* = 1.06	*w* = 1/[σ^2^(*F*_o_^2^) + (0.0739*P*)^2^ + 0.3774*P*] where *P* = (*F*_o_^2^ + 2*F*_c_^2^)/3
1901 reflections	(Δ/σ)_max_ < 0.001
209 parameters	Δρ_max_ = 0.39 e Å^−^^3^
1 restraint	Δρ_min_ = −0.18 e Å^−^^3^

### Special details

**Table:**

Geometry. Bond distances, angles *etc*. have been calculated using the rounded fractional coordinates. All su's are estimated from the variances of the (full) variance-covariance matrix. The cell e.s.d.'s are taken into account in the estimation of distances, angles and torsion angles
Refinement. Refinement on *F*^2^ for ALL reflections except those flagged by the user for potential systematic errors. Weighted *R*-factors *wR* and all goodnesses of fit *S* are based on *F*^2^, conventional *R*-factors *R* are based on *F*, with *F* set to zero for negative *F*^2^. The observed criterion of *F*^2^ > σ(*F*^2^) is used only for calculating -*R*-factor-obs *etc*. and is not relevant to the choice of reflections for refinement. *R*-factors based on *F*^2^ are statistically about twice as large as those based on *F*, and *R*-factors based on ALL data will be even larger.

### Fractional atomic coordinates and isotropic or equivalent isotropic displacement parameters (Å^2^)

**Table:**

	*x*	*y*	*z*	*U*_iso_*/*U*_eq_	
O1	0.28192 (10)	0.69820 (18)	0.43470 (13)	0.0184 (4)	
O2	−0.06046 (11)	0.61746 (18)	0.43776 (14)	0.0212 (4)	
O3	0.32497 (11)	0.31450 (18)	0.43000 (13)	0.0201 (4)	
O4	0.06177 (15)	0.4417 (3)	0.63635 (14)	0.0332 (6)	
C1	0.11167 (14)	0.6473 (2)	0.44797 (16)	0.0146 (5)	
C2	0.19662 (15)	0.7556 (2)	0.44266 (16)	0.0148 (5)	
C3	0.18243 (15)	0.9436 (2)	0.4447 (2)	0.0213 (6)	
C4	0.08584 (18)	0.9945 (3)	0.4923 (2)	0.0287 (7)	
C5	−0.00064 (16)	0.9004 (3)	0.4487 (2)	0.0282 (7)	
C6	0.01837 (15)	0.7154 (2)	0.44477 (17)	0.0170 (5)	
C7	0.12663 (14)	0.4583 (2)	0.44760 (16)	0.0143 (5)	
C8	0.16996 (17)	0.3960 (3)	0.35574 (16)	0.0169 (6)	
C9	0.1140 (2)	0.4019 (3)	0.27230 (18)	0.0245 (7)	
C10	0.1531 (2)	0.3453 (3)	0.18896 (19)	0.0309 (8)	
C11	0.2489 (3)	0.2819 (3)	0.18615 (19)	0.0337 (8)	
C12	0.3059 (2)	0.2744 (3)	0.26740 (19)	0.0273 (7)	
C13	0.26539 (18)	0.3314 (3)	0.35123 (17)	0.0189 (6)	
C14	0.28205 (18)	0.3342 (3)	0.51564 (16)	0.0175 (6)	
C15	0.35058 (18)	0.2726 (3)	0.59122 (19)	0.0247 (7)	
C16	0.3183 (2)	0.3350 (3)	0.6870 (2)	0.0312 (8)	
C17	0.2073 (2)	0.3126 (4)	0.70056 (19)	0.0329 (9)	
C18	0.14584 (19)	0.3913 (3)	0.62272 (17)	0.0226 (6)	
C19	0.18972 (17)	0.3968 (3)	0.52852 (15)	0.0159 (6)	
H2	−0.11220	0.67370	0.44770	0.0320*	
H3A	0.18270	0.98680	0.37960	0.0260*	
H3B	0.23900	0.99530	0.47850	0.0260*	
H4A	0.08960	0.96920	0.56020	0.0340*	
H4B	0.07550	1.11630	0.48490	0.0340*	
H5A	−0.06160	0.92160	0.48590	0.0340*	
H5B	−0.01220	0.94270	0.38430	0.0340*	
H7A	0.05940	0.40600	0.45460	0.0170*	
H9A	0.04840	0.44570	0.27360	0.0290*	
H10A	0.11430	0.34970	0.13320	0.0370*	
H11A	0.27580	0.24340	0.12840	0.0400*	
H12A	0.37160	0.23090	0.26570	0.0330*	
H15A	0.41910	0.31150	0.57810	0.0300*	
H15B	0.35100	0.14880	0.59120	0.0300*	
H16A	0.35440	0.27270	0.73640	0.0370*	
H16B	0.33550	0.45480	0.69320	0.0370*	
H17A	0.18780	0.36300	0.76130	0.0390*	
H17B	0.19210	0.19170	0.70360	0.0390*	

### Atomic displacement parameters (Å^2^)

**Table:**

	*U*^11^	*U*^22^	*U*^33^	*U*^12^	*U*^13^	*U*^23^
O1	0.0111 (6)	0.0182 (6)	0.0260 (8)	−0.0017 (5)	0.0003 (7)	0.0003 (7)
O2	0.0101 (7)	0.0176 (7)	0.0358 (9)	0.0000 (5)	−0.0006 (8)	−0.0019 (8)
O3	0.0144 (7)	0.0194 (7)	0.0265 (9)	0.0032 (5)	0.0007 (7)	0.0003 (7)
O4	0.0272 (10)	0.0447 (11)	0.0278 (10)	0.0020 (8)	0.0065 (8)	−0.0008 (8)
C1	0.0129 (9)	0.0127 (8)	0.0181 (10)	−0.0003 (7)	−0.0004 (8)	−0.0004 (8)
C2	0.0138 (9)	0.0145 (8)	0.0160 (9)	−0.0005 (7)	−0.0019 (8)	0.0003 (9)
C3	0.0165 (9)	0.0131 (8)	0.0343 (12)	−0.0026 (7)	0.0002 (10)	0.0005 (10)
C4	0.0197 (11)	0.0170 (10)	0.0493 (16)	−0.0001 (9)	0.0003 (11)	−0.0086 (11)
C5	0.0158 (10)	0.0142 (9)	0.0547 (17)	0.0031 (7)	−0.0026 (12)	−0.0034 (12)
C6	0.0153 (9)	0.0142 (8)	0.0214 (10)	0.0000 (7)	−0.0018 (9)	−0.0018 (9)
C7	0.0119 (8)	0.0119 (7)	0.0192 (10)	−0.0011 (7)	−0.0014 (8)	0.0009 (8)
C8	0.0220 (11)	0.0110 (10)	0.0177 (10)	−0.0013 (8)	−0.0006 (9)	−0.0005 (8)
C9	0.0336 (13)	0.0180 (11)	0.0218 (11)	−0.0016 (10)	−0.0075 (10)	0.0001 (9)
C10	0.0510 (17)	0.0207 (11)	0.0210 (12)	−0.0021 (11)	−0.0086 (12)	−0.0012 (10)
C11	0.0585 (19)	0.0233 (12)	0.0192 (12)	0.0008 (12)	0.0094 (12)	−0.0019 (10)
C12	0.0342 (14)	0.0179 (11)	0.0297 (13)	0.0035 (10)	0.0118 (11)	0.0002 (10)
C13	0.0235 (11)	0.0133 (10)	0.0198 (10)	−0.0012 (8)	0.0033 (9)	0.0005 (8)
C14	0.0198 (10)	0.0121 (9)	0.0207 (11)	−0.0017 (8)	−0.0039 (9)	0.0018 (8)
C15	0.0230 (12)	0.0209 (11)	0.0303 (13)	0.0016 (9)	−0.0069 (10)	0.0052 (10)
C16	0.0371 (15)	0.0303 (13)	0.0262 (13)	0.0039 (11)	−0.0113 (11)	0.0021 (11)
C17	0.0446 (17)	0.0358 (15)	0.0184 (12)	−0.0025 (12)	−0.0003 (11)	0.0070 (11)
C18	0.0266 (12)	0.0210 (10)	0.0201 (11)	−0.0048 (9)	0.0027 (10)	−0.0017 (9)
C19	0.0198 (10)	0.0119 (9)	0.0159 (10)	−0.0019 (8)	−0.0016 (8)	0.0013 (8)

### Geometric parameters (Å, º)

**Table:**

O1—C2	1.240 (2)	C14—C15	1.500 (3)
O2—C6	1.322 (2)	C15—C16	1.516 (4)
O3—C13	1.385 (3)	C16—C17	1.515 (4)
O3—C14	1.358 (3)	C17—C18	1.519 (4)
O4—C18	1.216 (3)	C18—C19	1.466 (3)
O2—H2	0.8400	C3—H3A	0.9900
C1—C6	1.368 (3)	C3—H3B	0.9900
C1—C7	1.526 (2)	C4—H4A	0.9900
C1—C2	1.436 (3)	C4—H4B	0.9900
C2—C3	1.517 (2)	C5—H5A	0.9900
C3—C4	1.520 (3)	C5—H5B	0.9900
C4—C5	1.518 (3)	C7—H7A	1.0000
C5—C6	1.503 (3)	C9—H9A	0.9500
C7—C19	1.513 (3)	C10—H10A	0.9500
C7—C8	1.516 (3)	C11—H11A	0.9500
C8—C9	1.407 (3)	C12—H12A	0.9500
C8—C13	1.385 (3)	C15—H15A	0.9900
C9—C10	1.375 (4)	C15—H15B	0.9900
C10—C11	1.385 (5)	C16—H16A	0.9900
C11—C12	1.389 (4)	C16—H16B	0.9900
C12—C13	1.389 (4)	C17—H17A	0.9900
C14—C19	1.351 (3)	C17—H17B	0.9900
			
C13—O3—C14	118.06 (18)	C2—C3—H3B	109.00
C6—O2—H2	109.00	C4—C3—H3A	109.00
C2—C1—C7	119.56 (16)	C4—C3—H3B	109.00
C6—C1—C7	121.03 (16)	H3A—C3—H3B	108.00
C2—C1—C6	119.14 (15)	C3—C4—H4A	110.00
O1—C2—C3	119.03 (17)	C3—C4—H4B	110.00
C1—C2—C3	119.85 (17)	C5—C4—H4A	110.00
O1—C2—C1	121.11 (15)	C5—C4—H4B	110.00
C2—C3—C4	112.44 (17)	H4A—C4—H4B	108.00
C3—C4—C5	109.8 (2)	C4—C5—H5A	109.00
C4—C5—C6	111.93 (19)	C4—C5—H5B	109.00
O2—C6—C5	116.77 (17)	C6—C5—H5A	109.00
C1—C6—C5	123.15 (17)	C6—C5—H5B	109.00
O2—C6—C1	120.08 (15)	H5A—C5—H5B	108.00
C1—C7—C19	113.18 (17)	C1—C7—H7A	107.00
C8—C7—C19	109.57 (17)	C8—C7—H7A	107.00
C1—C7—C8	112.30 (18)	C19—C7—H7A	107.00
C7—C8—C13	121.2 (2)	C8—C9—H9A	120.00
C9—C8—C13	118.0 (2)	C10—C9—H9A	120.00
C7—C8—C9	120.8 (2)	C9—C10—H10A	120.00
C8—C9—C10	120.9 (2)	C11—C10—H10A	120.00
C9—C10—C11	120.1 (3)	C10—C11—H11A	120.00
C10—C11—C12	120.3 (3)	C12—C11—H11A	120.00
C11—C12—C13	119.0 (3)	C11—C12—H12A	121.00
O3—C13—C12	115.9 (2)	C13—C12—H12A	120.00
C8—C13—C12	121.7 (2)	C14—C15—H15A	109.00
O3—C13—C8	122.3 (2)	C14—C15—H15B	109.00
O3—C14—C15	110.2 (2)	C16—C15—H15A	109.00
O3—C14—C19	123.7 (2)	C16—C15—H15B	109.00
C15—C14—C19	126.1 (2)	H15A—C15—H15B	108.00
C14—C15—C16	111.2 (2)	C15—C16—H16A	109.00
C15—C16—C17	111.0 (2)	C15—C16—H16B	109.00
C16—C17—C18	113.2 (2)	C17—C16—H16A	109.00
O4—C18—C19	120.7 (2)	C17—C16—H16B	109.00
C17—C18—C19	117.5 (2)	H16A—C16—H16B	108.00
O4—C18—C17	121.8 (2)	C16—C17—H17A	109.00
C7—C19—C18	118.8 (2)	C16—C17—H17B	109.00
C14—C19—C18	118.9 (2)	C18—C17—H17A	109.00
C7—C19—C14	122.2 (2)	C18—C17—H17B	109.00
C2—C3—H3A	109.00	H17A—C17—H17B	108.00
			
C14—O3—C13—C8	11.8 (3)	C8—C7—C19—C14	15.1 (3)
C14—O3—C13—C12	−166.1 (2)	C8—C7—C19—C18	−159.4 (2)
C13—O3—C14—C15	166.70 (19)	C7—C8—C9—C10	−179.9 (2)
C13—O3—C14—C19	−11.8 (3)	C13—C8—C9—C10	−0.1 (4)
C6—C1—C2—O1	−171.8 (2)	C7—C8—C13—O3	2.3 (3)
C6—C1—C2—C3	6.9 (3)	C7—C8—C13—C12	−179.8 (2)
C7—C1—C2—O1	2.4 (3)	C9—C8—C13—O3	−177.5 (2)
C7—C1—C2—C3	−178.9 (2)	C9—C8—C13—C12	0.3 (4)
C2—C1—C6—O2	171.6 (2)	C8—C9—C10—C11	−0.2 (4)
C2—C1—C6—C5	−8.3 (4)	C9—C10—C11—C12	0.3 (4)
C7—C1—C6—O2	−2.5 (4)	C10—C11—C12—C13	0.0 (4)
C7—C1—C6—C5	177.6 (2)	C11—C12—C13—O3	177.7 (2)
C2—C1—C7—C8	−65.0 (3)	C11—C12—C13—C8	−0.3 (4)
C2—C1—C7—C19	59.7 (3)	O3—C14—C15—C16	164.41 (19)
C6—C1—C7—C8	109.0 (2)	C19—C14—C15—C16	−17.2 (3)
C6—C1—C7—C19	−126.3 (2)	O3—C14—C19—C7	−2.7 (4)
O1—C2—C3—C4	−157.1 (2)	O3—C14—C19—C18	171.8 (2)
C1—C2—C3—C4	24.2 (3)	C15—C14—C19—C7	179.1 (2)
C2—C3—C4—C5	−52.2 (3)	C15—C14—C19—C18	−6.4 (4)
C3—C4—C5—C6	50.8 (3)	C14—C15—C16—C17	46.1 (3)
C4—C5—C6—O2	158.5 (2)	C15—C16—C17—C18	−54.0 (3)
C4—C5—C6—C1	−21.6 (3)	C16—C17—C18—O4	−151.9 (3)
C1—C7—C8—C9	−68.2 (3)	C16—C17—C18—C19	31.2 (3)
C1—C7—C8—C13	111.9 (2)	O4—C18—C19—C7	−3.0 (4)
C19—C7—C8—C9	165.1 (2)	O4—C18—C19—C14	−177.7 (2)
C19—C7—C8—C13	−14.7 (3)	C17—C18—C19—C7	173.9 (2)
C1—C7—C19—C14	−111.1 (2)	C17—C18—C19—C14	−0.8 (3)
C1—C7—C19—C18	74.5 (3)		

### Hydrogen-bond geometry (Å, º)

**Table:**

*D*—H···*A*	*D*—H	H···*A*	*D*···*A*	*D*—H···*A*
O2—H2···O1^i^	0.84	1.76	2.582 (2)	164
C5—H5*A*···O1^i^	0.99	2.42	3.034 (3)	119
C7—H7*A*···O2	1.00	2.35	2.822 (2)	108

Symmetry code: (i) *x*−1/2, −*y*+3/2, *z*.
